# Magnitude of unmet need for family planning and its predictors among reproductive age women in high fertility regions of Ethiopia: Evidence from Ethiopian Demographic and Health Survey

**DOI:** 10.1186/s12905-022-01982-w

**Published:** 2022-10-05

**Authors:** Desale Bihonegn Asmamaw, Wubshet Debebe Negash

**Affiliations:** 1 Department of Reproductive Health, Institute of Public Health, College of Medicine and Health Sciences, University of Gondar, Gondar, Ethiopia; 2 Department of Health Systems and Policy, Institute of Public Health, College of Medicine and Health Sciences, University of Gondar, Gondar, Ethiopia

**Keywords:** Unmet need for family planning, High fertility regions, Ethiopia

## Abstract

**Background:**

Unmet need for family planning refers to fertile women who want to limit or space their delivery but are not using contraceptive methods. Despite multiple studies were conducted to address family planning in Ethiopia, there is limited information on unmet need in high fertility regions. Knowing the magnitude and predictors of unmet need in the study area helps as an impute for interventions. Therefore, this study aims to assess the magnitude and predictors of unmet need for family planning among reproductive age women in high fertility regions of Ethiopia.

**Methods:**

A secondary data analysis was performed using the Ethiopian Demographic and Health Survey 2016. A total sample weight of 4312 currently married reproductive age women were included in this study. A multilevel mixed-effect binary logistic regression model was fitted. Finally, the odds ratios along with the 95% confidence interval were generated to determine the individual and community level factors of unmet need for family planning. A p-value less than 0.05 was declared as statistical significance.

**Results:**

The overall unmet need for family planning among currently married reproductive-age women in high fertility regions of Ethiopia was 29.78% (95% CI: 28.26, 31.3). Women with no formal education (AOR: 1.65, 95% CI: 1.17, 2.15), women in the poor wealth quantile (AOR: 1.67, 95% CI: 1.34, 2.09), women with no media exposure (AOR: 1.32, 95% CI: 1.09, 1.58), multiparous women (AOR: 1.57, 95% CI: 1.15, 2.16), sex of household head (AOR: 1.39, 95% CI: 1.11, 1.77) and rural residency (AOR: 2.45, 95% CI: 1.12, 3.59) were predictors of unmet need for family planning.

**Conclusion:**

The magnitude of unmet need for family planning among currently married reproductive-age women in high fertility regions of Ethiopia was high when compared to the national average and the United Nations sphere standard of unmet need for family planning. Education, wealth index, mass media, parity, sex of household head, and residence were independent predictors of unmet need for family planning among reproductive-age women in high fertility regions of Ethiopia. Any interventional strategies that reduce the unmet need for family planning should consider these factors to overcome the problems in the regions.

## Background

Unmet need for family planning (FP) defined by the World Health Organization (WHO) as “those who are fecund and sexually active but are not using any method of contraception, and report not wanting any more children or wanting to delay the next child” [1]. It is one of the indicators used to determine the achievement of universal reproductive health coverage [2]. Family planning is one aspect of women’s rights and constitutes the major feature of their reproductive health [3].

Globally, an estimated 214 million women have an unmet need for family planning services [4]. In developing countries, more than two hundred million women did not receive family planning services in 2012 [5]. In Ethiopia, the 2016 Ethiopia Demographic and Health Survey (EDHS) report reveals that 36% of currently married reproductive aged women (15–49) years use contraception and more than one in five married women have an unmet need for FP. With a total fertility rate of 4.6 [6]. The same report showed that the Somali, Afar, and Oromia regions of Ethiopia remained the highest total fertility rates with the fertility rate of 7.2, 5.5, and 5.4, respectively [6]. Studies elsewhere in Ethiopia have also revealed that the unmet need for family planning ranged from 16.2 to 37.4% [7–9].

As a result of unmet need for FP, high fertility, unintended pregnancy, and unsafe abortion are the main outcomes [10]. Approximately 80 million unintended pregnancies occur each year in the world. This in turn, has an effect on the mothers, children and the society at large [11]. For example, in low and middle income countries an estimated 18 million unsafe abortion takes place each year [12]. Although several interventions have been used to prevent unintended pregnancies, women, of low and middle-income countries are still affected by unplanned pregnancies [13, 14]. Failure to fulfill family planning also leads to short birth intervals this which results in both the newborn and the preceding child being at high risk of morbidity and mortality [15].

Religion, women’s age, number of living children, place of residence, respondent’s education, knowledge of family planning, respondent’s work status, being visited by a family planning worker, parity, and number of children at first use of contraceptives were factors associated with unmet need for family planning [7–9, 16, 17]. Existing studies also revealed that inaccessibility of family planning methods, lack of knowledge, religious belief, fear of side effects of contraceptives, and opposition by the husband were identified as reasons for not using contraceptives [18, 19].

Understanding the prevalence and predictors of unmet need for family planning helps each country to overcome the problem [20]. It is possible to prevent more than one million infant deaths and 54 million unwanted pregnancies by reducing the unmet need for FP [5]. Besides, it benefits a woman’s well-being, autonomy, and educational attainment. In Ethiopia, this study contributes to the field of family planning by identifying different predictors of unmet need for FP at the individual and community levels. The finding is also important for policymakers and program planners to realize women’s need for family planning in developing countries. Therefore, this research aimed to assess the magnitude and predictors of unmet needs in selected high fertility regions of Ethiopia. It is also important because it provides rational policy recommendations to help women meet their FP need.

## Methods

### Study design, setting, and period

The study was a cross-sectional analysis of data from recent EDHS, which was conducted by the Central Statistical Agency (CSA) in collaboration with the Federal Ministry of Health (FMoH) and the Ethiopian Public Health Institute (EPHI), which was a national representative sample conducted from January 18 to June 27, 2016. There are nine regional states in Ethiopia (Tigray, Afar, Amhara, Oromia, Benishangul, Gambela, South Nation, Nationalities and Peoples’ Region (SNNPR), Harari, and Somali), and two administrative cities (Addis Ababa and Dire-Dawa), 611 Districts, and 15,000 Kebeles. Afar, Somali and Oromia regions were included in this study. These regions were selected because they are the high fertility rate regions in Ethiopia with fertility rates above 5.0, a value that is higher than the rate of 4.6 in Ethiopia and 2.47 worldwide [6, 21].

The health care system in Ethiopia is structured in a three-tier system: primary, secondary, and tertiary levels of care. The primary level of care including primary hospitals, health centers, and health posts), The secondary level of care is delivered by general hospitals and the tertiary level of health care is given by specialized hospitals [22].

## Data source, sampling procedure, and study population

The study was conducted using the EDHS 2016 data by accessing the official DHS program database at www.measuredhs.com after obtaining permission via an online request by explaining the purpose of the study. A two-stage stratified cluster sampling technique was used to select populations using the 2007 Population and Housing Census (PHC) as a sampling frame. It has been stratified by separating the nine regional states and the two city administrations of Ethiopia, into urban and rural areas.

**Fig. 1:**
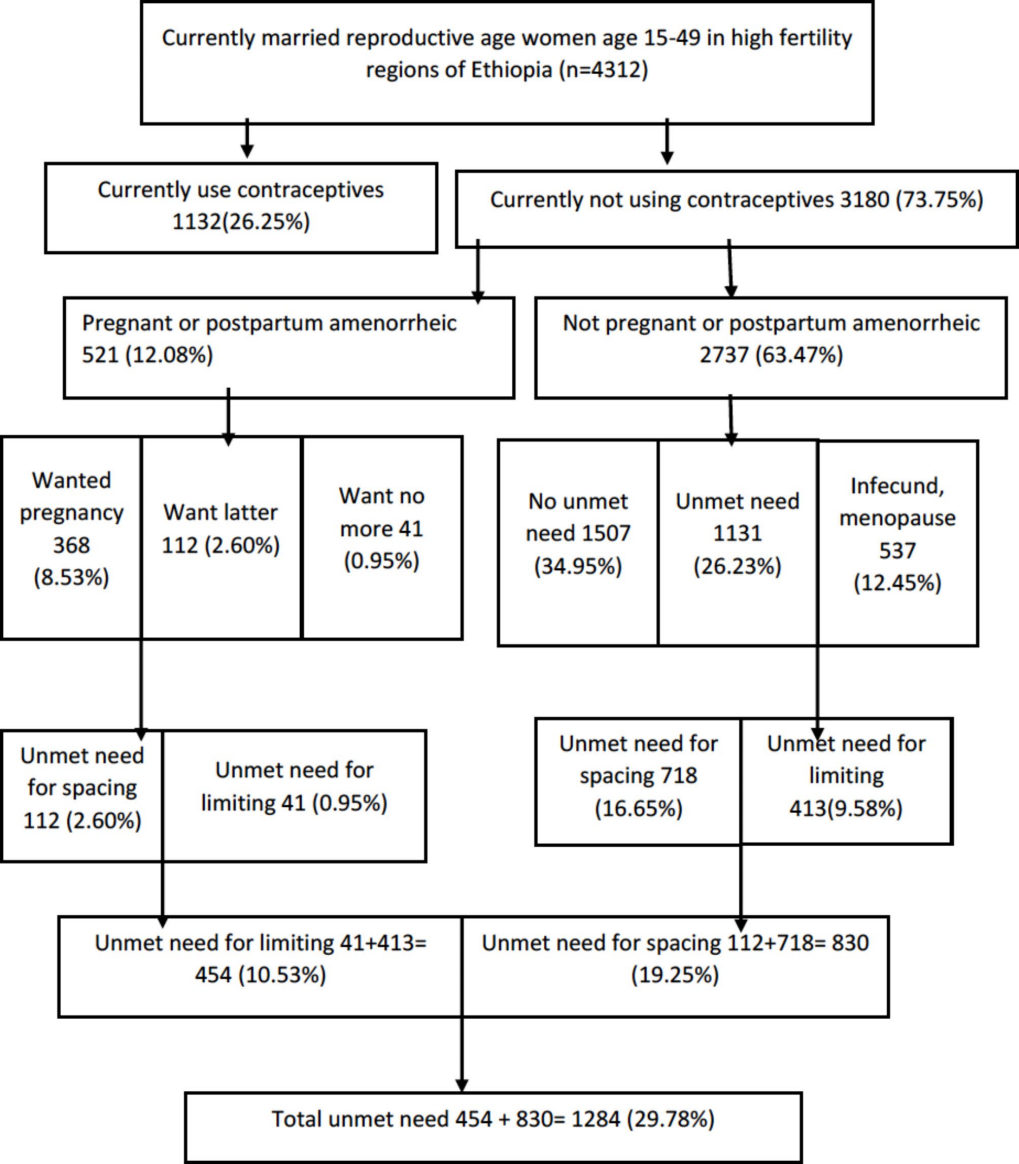
Unmet need for family planning among reproductive-age women in high fertility regions of Ethiopia (Westoff Model, 2012)

At the first stage, a total of 645 Enumeration Areas (EAs) (202 in urban areas and 443 in rural areas) proportional to EA size were selected, at the second stage, 28 households per cluster were selected with an equal probability of systematic selection [6]. For this study, we have used the women’s individual data set and the source population was currently married reproductive age women during the survey in the study regions (Afar, Somali, and Oromia). While the study population was all currently married reproductive age women who were in the selected EAs included in the analysis. Currently married reproductive age women who had never sex, and were not sexually active were excluded from this analysis. Data were collected by trained data collectors using pretested, structured, interviewer-administered questionnaires. A weighted sample of 4312 currently married reproductive age women was included in this study (Fig. 1).

## Measurements of variables

The dependent variable for the current study was unmet need for FP (yes/no). It is the sum of unmet need for spacing and limiting [23, 24]. Reproductive age women who were currently married, and or sexually active have unmet needs for family planning if they desire to space the next pregnancy or limit future pregnancy but not use any methods of family planning. Amenorrheic or pregnant women with unwanted or mistimed pregnancies or births who did not use family planning were also considered as unmet need for family planning [7, 24, 25].

Of the community level variables, residences (rural, urban) were directly accessed from DHS data sets. However, community level poverty and community-level media exposure were constructed by aggregating individual-level characteristics at the cluster level [26]. They were categorized as high or low based on the distribution of the proportion values generated for each community after checking the distribution by using the histogram. The aggregate variable was not normally distributed and the median value was used as a cut-off point for the categorization [26, 27].

### Data processing and analysis

Data were extracted from EDHS 2016 and cleaned, recoded, and analyzed using STATA version 14 Statistical Software. Throughout the analysis, sample weights were done to adjust for non-proportional allocation of the sample to strata and regions during the survey process and to restore the representative. Descriptive statistics were described using frequencies, percentages, median, and interquartile range, and were presented using tables, figures, and narratives. A multi-level logistic regression analysis was used after checking the model eligibility. It was assessed by calculating the intra-class correlation coefficient (ICC), and a model with an ICC greater than 10% was eligible for multilevel analysis. In this study, the ICC was 49%. Hence, the data were hierarchical (individuals were nested within the community).

First bi-variable multilevel logistic regression analysis was performed, and those variables with a p-value of < 0.20 were considered in the multivariable analysis. After selecting variables for multivariable multilevel analysis, four models were fitted; the null model (without independent variables), mode I (containing only individual-level factors), mode II (community-level factors), and model III (containing both individual and community level factors). Deviance was used to assess the model fitness, and the model with the lower deviance (model III) was the best-fitted model.

## **Result**s

## Socio-demographic characteristics of study participants

A total of 4312 weighted samples of reproductive age women in high fertility regions of Ethiopia were included. The median age of the study participants was 29 (IQR: 24, 36) years old. Nearly half (44.67%) of the study participants were under the age group of 25–34 years, 2806 (65.06%) of women had no formal education. Moreover, the majority (89%) of women were rural dwellers and 1868 (43.32%) of women fell in the poor wealth categories (Table 1).

**Table 1:** Socio-demographic and economic characteristics of study participants in high fertility region of Ethiopia

Variables	Frequency (n)	Percentage (%)
Age		
15–24	1054	24.44
25–34	1926	44.67
35–49	1332	30.89
Religion		
Orthodox	794	18.40
Muslim	2595	60.17
Protestant	774	17.94
Other	150	3.48
Resident		
Rural	3838	89
Urban	474	11
Region		
Afar	94	2.19
Somali	322	7.46
Oromia	3896	90.35
Educational status		
No formal education	2806	65.06
Primary education	1201	27.86
Secondary and above	305	7.08
Occupation of the respondent		
No employed	2526	58.58
Employed	1786	41.42
Household wealth index		
Poor	1868	43.32
Middle	834	19.35
Rich	1610	37.34
Media exposure		
Yes	1578	36.60
No	2734	63.40
Educational status of husband		
No formal education	2022	46.89
Primary education	1693	39.25
Secondary and above	597	13.86

## Obstetric history of the study participants

Of the total study participants, 3436 (79.70%) were multipara. More than half (51.05%) of women had ANC follow-up, and eight hundred and twenty-eight (73.12%) of participants decided jointly with their partners on contraceptives (Table 2).

**Table 2:** Obstetric history of the study participants in high fertility regions of Ethiopia

Variables	Frequency (n)	Percentage (%)
Parity		
Primipara	875	20.30
Multipara	3436	79.70
ANC follow up		
Yes	1631	51.05
No	1563	48.95
Terminated pregnancy		
Yes	426	9.88
No	3886	90.12
Desired number of children		
Have another	2345	54.37
Undecided	302	7.00
Wants no more	1666	38.62
Decision maker on contraceptive		
Mainly respondent	189	16.66
Mainly husband	116	10.25
Jointly	828	73.12
Number of living children		
No child	329	7.64
1–2	1991	27.63
3–4	1150	26.66
5+	1642	38.07

## The magnitude of unmet need for family planning

**Fig. 2:**
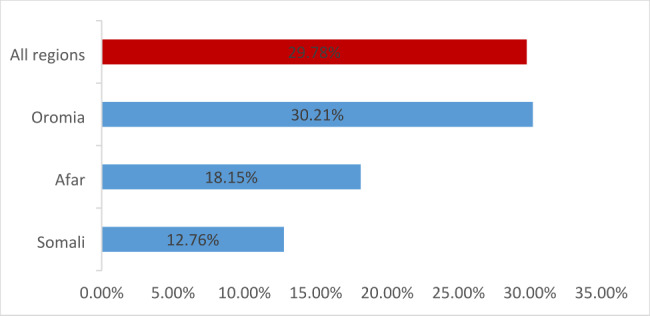
Unmet need for family planning in high fertility regions of Ethiopia

The magnitude of unmet need for FP in high fertility regions of Ethiopia was 29.78% (95% CI: 28.26, 31.30), with Oromia recording the highest prevalence of 30.21% (Fig. 2).

## Random effects and model fitness

The intra-class correlation (ICC) in the null model indicated that 49% of the overall variability of unmet need for FP was attributed to cluster variability. The median odds ratio for unmet need for FP was 4.52 in the null model, which indicates that there was a variation in unmet need for FP between clusters. This means that if we randomly selected individuals from different clusters, those in the cluster with the highest unmet need for FP had a 4.52 times higher chance of having an unmet need for FP than those in the cluster with the lowest unmet need for FP.

The proportional change in variance (PCV) also increased from 32.81% in the model I to 43.48% in the model III (a model with individual and community level variables), indicating that the final model (model III) best describes the variability of unmet need for FP. Deviance was also used to assess model fitness. The best fitting model was model III, which had the lowest deviance (Table 3).

**Table 3:** Model comparison and random effect analysis result in high fertility regions of Ethiopia

Random effect	Null model	Model 1	Model 2	Model 3
ICC	0.49	0.37	0.31	0.28
Variance	2.53	1.70	1.58	1.43
MOR	4.52	3.46	3.24	3.13
PCV (%)	Reference	32.81	37.55	43.48
**Model fitness**				
Deviance(-2 LL)	2417.78	2357.90	2246.62	2204.34

## Predictors of unmet need for family planning

In the final model (model III), after adjusting for individual and community level factors, individual-level variables such as education, wealth quintile, media exposure, and parity, sex of household head as well as community-level variables such as residency, were identified as predictors of unmet need for FP among reproductive-age women.

Accordingly, the odds of unmet need for FP among women with no formal education were 1.65 times higher than those women with formal education (AOR: 1.65, 95% CI: 1.17, 2.15). Women who had no media exposure had 1.32 times more odds of having an unmet need for FP than those who had media exposure (AOR: 1.32, 95% CI: 1.09, 1.58). Multiparous women were found to be 1.57 times more likely to have had an unmet need for family planning compared with those women with primipara (AOR: 1.57, 95% CI: 1.15, 2.88). The odds of unmet need for FP among women who live in rural areas were 2.45 times (AOR: 2.45, 95% CI: 1.12, 3.59) more likely than among women who live in urban areas. The odds of unmet need for FP from households with poor wealth status were 1.67 times (AOR: 1.67, 95% CI: 1.34, 2.09) higher than women from rich households’ wealth status (Table 4). Those households lead by male were 1.39 times (AOR = 1.39; 95% CI : 1.11, 1.77) more likely experienced unmet need for family planning as compare to counterpart.

**Table 4:** Multi-level mixed-effect logistic regression analysis predictors of unmet need for FP among reproductive age women in high fertility regions of Ethiopia

Variables	Model 1 AOR (95% CI)	Model 2 AOR (95%CI)	Model 3 AOR (95%CI)
**Individual-level** **Characteristics**			
Age			
15–24	1.31 (0.97, 1.76)		1.09 (0.69, 1.74)
25–34	1.24 (0.95,1.61)		0.79 (0.59, 1.06)
35+	1		1
Educational status of the respondents			
No formal education	1.23 (1.03, 1.71)		1.65 (1.17, 2.15)
Primary education	1.21 (0.80, 1.83)		1.35 (0.79, 2.85)
Secondary and higher	1		1
Wealth index			
Poor	1.54(1.25, 1.91)		1.67 (1.34, 2.09)
Middle	1.13 (0.89, 1.42)		1.11 (0.89, 1.41)
Rich	1		1
Media exposure			
No	1.30(1.08, 1.56)		1.32 (1.09, 1.58)
Yes	1		1
Number of alive children			
No child	1.02 (0.71, 1.25)		1.05 (0.43, 2.43)
1–2	0.78 (0.62 1.10)		0.73 (0.59, 1.04)
3–4	0.81 (0.63, 1.12)		0.79 (0.65, 1.09)
5+	1		1
Parity			
Prime para	1		1
Multipara	1.55 (1.14, 2.13)		1.57(1.15, 2.16)
Sex of household head			
Male	1.22 (0.96,1.53)		1.39 (1.11, 1.77)
Female	1		1
**Community-level variables**			
Community-level poverty			
High		0.97 (0.63,1.47)	0.79 (0.53, 1.19)
Low		1	1
Community media exposure			
High		0.81 (0.56, 1.17)	0.87 (0.62, 1.25)
Low		1	1
Residency			
Rural		2.19 (1.34, 3.59)	2.45 (1.12, 3.59)
Urban		1	1
Region			
Somali		0.64 (0.0.31, 1.30)	0.54 (0.27, 1.11)
Oromia		`1.76 (0.91, 3.41)	1.88 (0.97, 3.64)
Afar		1	1
Distance to the health facility			
Big problem		1.19 (0.97, 1.44)	1.13 (0.92, 1.38)
Not big problem		1	1

* Statistically significant at p-value < 0.05, AOR Adjusted Odds Ratio, COR Crude Odds Ratio, Null

Model: adjusted for individual-level characteristics, Model 2: adjusted for community-level

Characteristics, Model 3: adjusted for both individual and community-level characteristics

## Discussion

This study was conducted to examine the magnitude and predictors of the unmet need for family planning among currently married reproductive age women in high fertility regions of Ethiopia. In the current study, the unmet need for FP was found to be 29.78% in high fertility regions, with Oromia having the highest prevalence of 30.21%. The result of the study showed that level of education, wealth quintile, media exposure, parity, sex of household head, and residency were identified as the predictive factors for unmet need for FP among currently married reproductive age women in high fertility region of Ethiopia.

The magnitude of unmet need for FP in the current study is higher than the national magnitude, which was 22% [6]. And significantly higher than previous regional studies in Ethiopia [8, 23, 28]. It is higher from the national target of reducing the level of unmet need for FP to 10% by 2020 [29]. The magnitude was higher compared with the United Nations sphere standard of unmet need for FP, which is considered high if greater than 25%, and the global estimate of unmet need for FP reproductive age women (24.3%) [27, 30]. The finding was also higher than studies conducted in Zambia (21%) [31], and Bangladesh (13.50%) [24]. The higher magnitude of unmet need for FP in this study might be due to the fact that the current study was conducted in high fertility regions of Ethiopia; those regions are developing regions; the pastoralist community of Ethiopia; has limited access to health facilities; low access to contraceptives; low awareness of modern contraceptive methods; poor infrastructure; and husbands are strongly opposed their wife to use family planning [32–34]. Furthermore, in the study areas where most of them are uneducated, Muslim religion followers and the culture in those regions are not allowed to use family planning even if the women wanted to use it. Members of the Muslim religion are strongly opposed to contraceptive use [35, 36].

However, the magnitude of unmet need for FP in the present study was lower than in the study conducted in India [37]. This variation could be attributed to the difference in the target population. The current study was done among reproductive age women while the study conducted in India was done among young women. The evidences showed that usually, young women are far from adequately meeting the needs of family planning and lack the knowledge, agency, or resources to make decisions regarding their reproduction [38, 39]. It suggests that even though there was an improvement in contraceptive prevalence among currently married reproductive age women in the regions; achieving the target and maximizing the benefits of contraception requires dedication to provide contraceptives to those women with the identified unmet need.

In this study, women with no formal education were more likely to have an unmet need for FP as compared to women with formal education. The finding of this study is in agreement with those of studies conducted in Ethiopia [7, 32, 33], Nigeria [40], and Malawi [41]. The possible reason might be that women with no formal education are less likely to be exposed to FP through the media and other ways of exposure, which compromise access to contraceptives and have no easy to understand the health benefits of the contraceptive in reducing fertility, unintended pregnancy, unsafe abortion, and other maternal and child problems [42, 43]. In addition, non-educated women are less likely to be empowered, which subsequently increases their unmet need [42]. This suggests that education will be one way to reduce the unmet need for contraceptives in these high fertility regions.

The likelihood of the unmet need for FP among women from households with poor wealth quintile was higher than those from households with rich wealth quintile. This finding is supported by studies done in Ethiopia [25, 44], Nigeria [45], and developing countries [46]. The reason might be that women from poor households cannot be able to deal with the cost barrier associated with access to contraceptive use as compared to those from rich households since they cannot be able to overcome both the direct and indirect costs associated with contraceptive uptake [47]. Another possible reason could be that as income decreases, exposure to different types information and the financial accessibility of services will be compromised [48].

Another factor that predicts the unmet need for FP found in our study was media exposure; A woman who had no media exposure was 1.53 times more likely to have an unmet need for family planning as compared to those who had media exposure. The finding is consistent with studies done in Mali [49], and Nigeria [50], where exposure to mass media has a substantial positive effect on contraceptive use and intended future use of contraception. The reason for this may be that women with no media exposure might not have a better understanding of contraception, which cannot have a positive change in their attitude toward contraception [50, 51]. The study indicates that media exposure will reduce the barriers to access and use of health care services, including contraception.

According to this study, the odds of having an unmet need for FP among reproductive age women living in rural areas were 6.16 times higher than their counterparts. This is similar to studies conducted in Ethiopia [52], Nigeria [53], and Bangladesh [54]. This might be due to a variety of reasons; in Ethiopia; rural residents have poor health service accessibility and low awareness of contraceptives due to the fact that rural women are less educated, have limited access to mass media, have insufficient income, and poor infrastructure, which has a positive impact on modern contraceptive use [6, 44]. Moreover, evidence revealed a high concentration of sexual and reproductive health services delivery in an urban area in Ethiopia [23, 27, 55]. The odds of unmet need for family planning were higher among male-headed households as compare to female-headed house. This finding is in line with study conducted in Nigeria [45] and East Africa [56]. This might be due to the female headed household may increase resource gain and control [57].

In this study, parity was found to be one of the predictors of unmet need for FP. Multipara women were 1.77 times more likely to have unmet needs for family planning when compared to their counterparts. This is congruent with the results of other studies [7, 23], which revealed multipara women were more likely to use family planning compared to primipara women. The possible explanation might be that the more children the woman is having; the more likely she wants to space or limit the number of children she will have [7, 33]. This indicates that the more she uses family planning, the more she has an unmet need.

The main strength of this study was that it used nationally representative survey data and focused on high fertility regions in Ethiopia. In addition, the DHS uses validated instruments in its appraisals of datasets along with the large sample size and well-designed procedures, such as training field enumerators and employing well-tested methods for data collection. However, since the surveys are cross-sectional design, causality cannot be established for the findings. Additionally, due to the use of secondary data, essential factors like attitude toward family planning methods, husband perspective on FP, and socio-cultural factors were not available in the EDHS data set. Hence, it was not possible to incorporate these variables.

## Conclusion

In the current study, the magnitude of unmet need for family planning among currently married reproductive-age women in high fertility regions of Ethiopia was high when compared to the national average and the United Nations sphere standard of unmet need for family planning. Education, wealth index, mass media, parity, sex of household head and residence were independent predictors of unmet need for family planning among reproductive-age women in high fertility regions of Ethiopia. Any interventional strategies that reduce the unmet need for family planning should consider these factors to overcome the problems in the regions. Future researchers interested in this area should also consider qualitative variables such as socio-cultural factors, which might have a great effect on the unmet need for family planning.

## Data Availability

This study used data from the 2016 Ethiopian Demographic and Health Survey, which is freely available online at (https://www.dhsprogram.com).

## Abbreviations

AOR: Adjusted Odds Ratio.
CI: Confidence interval.
EA: Enumeration Areas.
EDHS: Ethiopia Demographic and Health Survey.
FP: Family Planning.
ICC: Intra-class Correlation Coefficient.
MOR: Median Odds Ratio.
PCV: Proportional Change in Variance.
SD: Standard Deviation.
SSA: Sub-Saharan Africa.
WHO: World Health Organization.

## References

[1] WHO. **Unmet need for family planning**. *internet* 2021.

[2] Alkema L, Kantorova V, Menozzi C, Biddlecom A (2013). National, regional, and global rates and trends in contraceptive prevalence and unmet need for family planning between 1990 and 2015: a systematic and comprehensive analysis. The Lancet.

[3] Behrman JA, Wright KQ, Grant MJ, Soler-Hampejsek E: **Trends in Modern Contraceptive Use among Young Adult Women in sub-Saharan Africa** 1990 **to 2014**. *Studies in family planning* 2018, **49**(4):319–344. 10.1111/sifp.12075 30431643

[4] Sedgh G, Ashford LS, Hussain R: **Unmet need for contraception in developing countries**: **examining women**’**s reasons for not using a method**. 2016.

[5] Singh S, Darroch JE: **Adding it up**: **costs and benefits of contraceptive services—estimates for** 2012. 2012.

[6] Csa I: **Central statistical agency** (**CSA**)[**Ethiopia] and ICF**. *Ethiopia demographic and health survey, Addis Ababa, Ethiopia and Calverton, Maryland, USA* 2016.

[7] Hailemariam A, Haddis F (2011). Factors affecting unmet need for family planning in southern nations, nationalities and peoples region, Ethiopia. Ethiop J health Sci.

[8] Tadele A, Abebaw D, Ali R (2019). Predictors of unmet need for family planning among all women of reproductive age in Ethiopia. Contracept reproductive Med.

[9] Worku SA, Ahmed SM, Mulushewa TF: **Unmet need for family planning and its associated factor among women of reproductive age in Debre Berhan Town**, **Amhara**, **Ethiopia**. *BMC research notes* 2019, **12**(1):1–6. 10.1186/s13104-019-4180-9 PMC6419818 30876437

[10] Shifa GT, Kondale M (2014). High unmet need for family planning and factors contributing to it in southern Ethiopia: A community based cross-sectional study. Global J Med Res.

[11] Elweshahi HMT, Gewaifel GI, Sadek SSE-D, El-Sharkawy OG (2018). Unmet need for postpartum family planning in Alexandria, Egypt. Alexandria J Med.

[12] Stover J, Winfrey W (2017). The effects of family planning and other factors on fertility, abortion, miscarriage, and stillbirths in the Spectrum model. BMC Public Health.

[13] Hindin MJ, Kalamar AM, Thompson T-A, Upadhyay UD (2016). Interventions to prevent unintended and repeat pregnancy among young people in low-and middle-income countries: a systematic review of the published and gray literature. J Adolesc Health.

[14] Berta M, Feleke A, Abate T, Worku T, Gebrecherkos T (2018). Utilization and associated factors of modern contraceptives during extended postpartum period among women who gave birth in the last 12 months in Gondar Town, Northwest Ethiopia. Ethiop J health Sci.

[15] Bongaarts J: **United Nations Department of Economic and Social Affairs**, **Population Division World Family Planning 2020**: **Highlights**, **United Nations Publications**, 2020. **46 p**. In.: Wiley Online Library; 2020.

[16] Mekonnen W, Worku A (2011). Determinants of low family planning use and high unmet need in Butajira District, South Central Ethiopia. Reproductive health.

[17] Ali AAA, Okud A (2013). Factors affecting unmet need for family planning in Eastern Sudan. BMC Public Health.

[18] Khalil SN, Alzahrani MM, Siddiqui AF (2018). Unmet need and demand for family planning among married women of Abha, Aseer Region in Saudi Arabia. Middle East Fertility Society Journal.

[19] Wulifan JK, Brenner S, Jahn A, De Allegri M (2015). A scoping review on determinants of unmet need for family planning among women of reproductive age in low and middle income countries. BMC Womens Health.

[20] Ashford L. Unmet need for family planning: Recent trends and their implications for programs. Population Reference Bureau Washington, DC; 2003.

[21] **African countries with the highest fertility rate** | **Statista https**://**worldpopulationreview**.**com**/**countries**/**total**-**fertility**-**rate**. cited on December 8, 2021.

[22] MOH E: **Federal Democratic Republic of Ethiopia Ministry of Health Health Sector Development Program IV October 2010 Contents**. *October* 2010 2014.

[23] Solomon T, Nigatu M, Gebrehiwot TT, Getachew B (2019). Unmet need for family planning and associated factors among currently married reproductive age women in Tiro Afeta district, South West Ethiopia, 2017: cross-sectional study. BMC Womens Health.

[24] Bishwajit G, Tang S, Yaya S, Feng Z (2017). Unmet need for contraception and its association with unintended pregnancy in Bangladesh. BMC Pregnancy Childbirth.

[25] Yalew M, Adane B, Kefale B, Damtie Y (2020). Individual and community-level factors associated with unmet need for contraception among reproductive-age women in Ethiopia; a multi-level analysis of 2016 Ethiopia Demographic and Health Survey. BMC Public Health.

[26] Liyew AM, Teshale AB (2020). Individual and community level factors associated with anemia among lactating mothers in Ethiopia using data from Ethiopian demographic and health survey, 2016; a multilevel analysis. BMC Public Health.

[27] Getaneh T, Negesse A, Dessie G, Desta M, Moltot T (2020). Predictors of unmet need for family planning in Ethiopia 2019: a systematic review and meta analysis. Archives of Public Health.

[28] Gebre G, Birhan N, Gebreslasie K. **Prevalence and factors associated with unmet need for family planning among the currently married reproductive age women in Shire-Enda-Slassie, Northern West of Tigray, Ethiopia 2015: a community based cross-sectional study**. Pan African Medical Journal 2016, 23(1). 10.11604/pamj.2016.23.195.8386 PMC4907757 27347284

[29] Ethiopia F. **Health Sector Transformation Plan (HSTP): 2015/16-– 2019/20**. In. Addis Ababa; 2015.

[30] Kantorová V, Wheldon MC, Ueffing P, Dasgupta AN (2020). Estimating progress towards meeting women’s contraceptive needs in 185 countries: A Bayesian hierarchical modelling study. PLoS Med.

[31] Mulenga JN, Bwalya BB, Mulenga MC, Mumba K. **Determinants of unmet need for family planning among married women in Zambia**. Journal of Public Health in Africa 2020, 11(1). 10.4081/jphia.2020.1084 PMC7649728 33209230

[32] Tegegne TK, Chojenta C, Forder PM, Getachew T, Smith R, Loxton D (2020). Spatial variations and associated factors of modern contraceptive use in Ethiopia: a spatial and multilevel analysis. BMJ open.

[33] Girma Garo M, Garoma Abe S, Dugasa Girsha W, Daka DW (2021). Unmet need for family planning and associated factors among currently married women of reproductive age in Bishoftu town, Eastern Ethiopia. PLoS ONE.

[34] Demissie DB, Kurke A, Awel A, Oljira K: **Male involvement in family planning and associated factors among Marriedin Malegedo town west Shoa zone**, **Oromia**, **Ethiopia**. *planning* 2016, **15**.

[35] Wafula SW (2015). Regional differences in unmet need for contraception in Kenya: insights from survey data. BMC Womens Health.

[36] Gebre-Egziabhere T (2018). Emerging Regions in Ethiopia: Are they catching up with the rest of Ethiopia?. East Afr Social Sci Res Rev.

[37] Yadav K, Agarwal M, Shukla M, Singh JV, Singh VK (2020). Unmet need for family planning services among young married women (15–24 years) living in urban slums of India. BMC Womens Health.

[38] Prata N, Weidert K, Sreenivas A (2013). Meeting the need: youth and family planning in sub-Saharan Africa. Contraception.

[39] Glinski A, Sexton M, Petroni S: **Adolescents and family planning**: **what the evidence shows**. **Washington** (**DC**): **International Center for Research on Women**; 2014. In.; 2017.

[40] Solanke BL, Oyinlola FF, Oyeleye OJ, Ilesanmi BB (2019). Maternal and community factors associated with unmet contraceptive need among childbearing women in Northern Nigeria. Contracept reproductive Med.

[41] Nkoka O, Mphande WM, Ntenda PA, Milanzi EB, Kanje V, Guo SJ (2020). Multilevel analysis of factors associated with unmet need for family planning among Malawian women. BMC Public Health.

[42] Samarakoon S, Parinduri RA (2015). Does education empower women? Evidence from Indonesia. World Dev.

[43] Riaz S, Pervaiz Z (2018). The impact of women’s education and employment on their empowerment: an empirical evidence from household level survey. Qual Quant.

[44] Alem AZ, Agegnehu CD (2021). Magnitude and associated factors of unmet need for family planning among rural women in Ethiopia: a multilevel cross-sectional analysis. BMJ open.

[45] Oginni AB, Ahonsi BA, Adebajo S (2015). Trend and determinants of unmet need for family planning services among currently married women and sexually active unmarried women aged 15–49 in Nigeria (2003–2013). Afr Popul Stud.

[46] Gwatkin DR, Rutstein S, Johnson K, Suliman E, Wagstaff A, Amouzou A. **Socio-economic differences in health, nutrition, and population within developing countries**. Washington, DC: World Bank 2007, 287. 18293634

[47] Hulme J, Dunn S, Guilbert E, Soon J, Norman W (2015). Barriers and facilitators to family planning access in Canada. Healthc Policy.

[48] Alaba OO, Olubusoye OE, Olaomi J (2015). Spatial pattern and determinants of unmet need of family planning in Nigeria. South Afr Family Pract.

[49] Zamawe CO, Banda M, Dube AN (2016). The impact of a community driven mass media campaign on the utilisation of maternal health care services in rural Malawi. BMC Pregnancy Childbirth.

[50] Ajaero CK, Odimegwu C, Ajaero ID, Nwachukwu CA (2016). Access to mass media messages, and use of family planning in Nigeria: a spatio-demographic analysis from the 2013 DHS. BMC Public Health.

[51] Retherford RD, Mishra VK: **Media exposure increases contraceptive use**. 1997. 12293013

[52] Gebre MN, Edossa ZK (2020). Modern contraceptive utilization and associated factors among reproductive-age women in Ethiopia: evidence from 2016 Ethiopia demographic and health survey. BMC Womens Health.

[53] Johnson OE (2017). Determinants of modern contraceptive uptake among Nigerian women: evidence from the national demographic and health survey. Afr J Reprod Health.

[54] Haq I, Sakib S, Talukder A (2017). Sociodemographic factors on contraceptive use among ever-married women of reproductive age: evidence from three demographic and health surveys in Bangladesh. Med Sci.

[55] Genet E, Abeje G, Ejigu T (2015). Determinants of unmet need for family planning among currently married women in Dangila town administration, Awi Zone, Amhara regional state; a cross sectional study. Reproductive health.

[56] Alie MS, Abebe GF, Negesse Y (2022). Magnitude and determinants of unmet need for family planning among reproductive age women in East Africa: multilevel analysis of recent demographic and health survey data. Contracept Reproductive Med.

[57] Kareem YO, Morhason-Bello IO, OlaOlorun FM, Yaya S (2021). Temporal relationship between Women’s empowerment and utilization of antenatal care services: lessons from four National Surveys in sub-Saharan Africa. BMC Pregnancy Childbirth.

